# Quantitative relationship between the octanol/water partition coefficient and the diffusion limitation of the exchange between adipose and blood

**DOI:** 10.1186/1472-6904-10-1

**Published:** 2010-01-07

**Authors:** David G Levitt

**Affiliations:** 1 Department of Integrative Biology and Physiology, University of Minnesota, 6-125 Jackson Hall, 321 Church St. S. E., Minneapolis, MN 55455, USA

## Abstract

**Background:**

The goal of physiologically based pharmacokinetics (PBPK) is to predict drug kinetics from an understanding of the organ/blood exchange. The standard approach is to assume that the organ is "flow limited" which means that the venous blood leaving the organ equilibrates with the well-stirred tissue compartment. Although this assumption is valid for most solutes, it has been shown to be incorrect for several very highly fat soluble compounds which appear to be "diffusion limited". This paper describes the physical basis of this adipose diffusion limitation and its quantitative dependence on the blood/water (K_bld-wat_) and octanol/water (K_ow_) partition coefficient.

**Methods:**

Experimental measurements of the time dependent rat blood and adipose concentration following either intravenous or oral input were used to estimate the "apparent" adipose perfusion rate (F_A_) assuming that the tissue is flow limited. It is shown that the ratio of F_A _to the anatomic perfusion rate (F) provides a measure of the diffusion limitation. A quantitative relationship between this diffusion limitation and K_bld-wat _and K_ow _is derived. This analysis was applied to previously published data, including the Oberg et. al. measurements of the rat plasma and adipose tissue concentration following an oral dose of a mixture of 13 different polychlorinated biphenyls.

**Results:**

Solutes become diffusion limited at values of log K_ow _greater than about 5.6, with the adipose-blood exchange rate reduced by a factor of about 30 for a solute with a log K_ow _of 7.36. Quantitatively, a plot of F_A_/F versus K_ow _is well described assuming an adipose permeability-surface area product (PS) of 750/min. This PS corresponds to a 0.14 micron aqueous layer separating the well-stirred blood from the adipose lipid. This is approximately equal to the thickness of the rat adipose capillary endothelium.

**Conclusions:**

These results can be used to quantitate the adipose-blood diffusion limitation as a function of K_ow_. This is especially important for the highly fat soluble persistent organic chemicals (e.g. polychlorinated biphenyls, dioxins) whose pharmacokinetics are primarily determined by the adipose-blood exchange kinetics.

## Background

Physiologically Based Pharmacokinetics (PBPK) refers to the approach of modeling drug kinetics using a realistic physiological description of the animal [1,2]. A central feature of this approach is the quantitative description of the tissue-blood exchange. The most basic approach (and the standard one) is to assume that this exchange is "flow limited" - that is, the venous blood leaving the capillary has equilibrated with the well mixed tissue space. For the flow limited model this tissue-blood exchange depends on only two parameters: a) the tissue perfusion rate (kg/min/kg); and b) the blood/tissues partition coefficient. This model has the major advantage that if one has determined the perfusion rate using one solute, the tissue-blood kinetic exchange can be predicted for any other solute for which the blood/tissue partition is known.

There are a number of solutes for which this flow limited model is clearly not valid and for which a "diffusion limited" model must be used. One such class are the large (e.g. inulin) or highly protein bound (e.g. dicloxacillin and ceftriaxone) extracellular solutes which have a significant capillary permeability limitation [3]. Similarly, a number of water soluble molecules such as actinomycin-D and methotrexate have been shown to have cell membrane limited uptake [4]. It is not surprising that these highly water soluble solutes with their low cell membrane permeability might be diffusion limited. These solutes represent relatively rare exceptions and the great majority of solutes that are used in PBPK modeling are highly lipid soluble with high (nearly infinite) cell membrane permeability and the predictions of the flow limited PBPK model generally provides good agreement with the experimental pharmacokinetics.

However, there are some notable exceptions to this general rule where the adipose-blood exchange of several highly lipid soluble molecules seems to be diffusion limited [5-7]. The purpose of this paper is to provide a detailed mechanistic analysis of the origin of the diffusion limitation of these highly lipid soluble molecules. It will be shown that the magnitude of the diffusion limitation can be directly related to and predicted by the lipid/water partition coefficient. This analysis provides general criteria for predicting the degree of adipose tissue diffusion limitation just from knowledge of the lipid/water (or octanol/water, see below) partition coefficient. This is the first detailed discussion of the quantitative relationship between lipid/water partition and diffusion limitation that I am aware of.

This analysis is especially important for the persistent organic pollutants (e.g. dioxins and polychlorinated biphenyls) whose pharmacokinetics are dominated by the kinetics of adipose-blood exchange [8]. The use of PBPK model predictions is essential for this solute class because it is not possible to accurately measure their pharmacokinetics in humans. As the following analysis shows, the adipose-blood exchange rate for the most highly lipid soluble solutes can be as much as 30 times slower than is predicted assuming flow limited kinetics.

The approach described here is based on an analysis of experimental measurements of the "apparent" adipose perfusion rate (F_A_). The "apparent" rate is the perfusion rate that would be predicted assuming that the exchange is blood flow limited. It will be shown that the ratio of this "apparent" rate and the true anatomic adipose perfusion rate (F_A_/F) can be used to quantitate the degree of diffusion limitation.

## Methods

### Dependence of the adipose-blood exchange rate (Clr) on the intrinsic capillary permeability-surface area product (PS) and the blood-water partition coefficient (K_bld-wat_)

**Figure 1:**
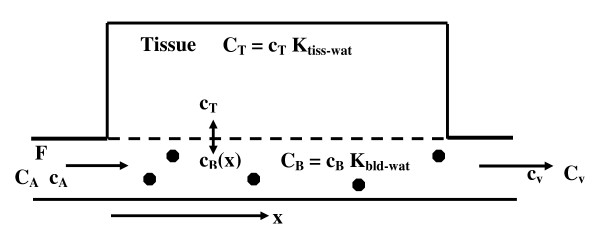
**Schematic diagram of the concentrations in capillary blood and adipose tissue and the factors involved in the tissue-blood solute exchange**. The upper case letters indicate the absolute blood (C_B_, C_A_, C_V_) or tissue (C_T_) concentration while the lower case (c_B_, c_T_) indicates the free aqueous blood (c_B_) or tissue (c_T_) concentration.

Figure 1 shows a schematic diagram of the factors involved in the solute exchange between the tissue and blood. The upper case letters in Figure 1 indicate the absolute blood (C_B_, C_A_, C_V_) or tissue (C_T_) concentration while the lower case indicates the free aqueous blood (c_B_) or tissue (c_T_) concentration. It is this free aqueous concentration that determines the diffusional exchange rate between the blood and tissue. The capillary concentration varies as a function of the distance (x) from the arterial end of the capillary. It is assumed that the tissue compartment is well mixed and can be represented by an average value that does not depend on x.

It is assumed that the tissue consists of N capillaries/cm^3^, all with exactly the same geometry, blood flow, permeability, etc. It is also assumed that the relation between the capillary and tissue concentration is in a pseudo steady state. The steady state differential equation for the concentration in the capillary as a function of position is(1)

where F is the tissue perfusion rate (kg/min/kg), P is the intrinsic capillary permeability defined in terms of the free water concentration (cm/min), N is the number of capillaries per cm^3 ^tissue, and a is the capillary radius (cm). The total blood concentration C_B _can be related to the free water concentration (c_B_) using the blood/water partition coefficient (C_B _= K_bld-wat _c_B_). Integrating eq [1] over the length of the capillary (L) and solving for the venous concentration leaving the capillary:(2)

where PS (min^-1^) is the permeability-surface area product per tissue weight (S = 2πaNL). Equation [2] can be related to the fractional clearance or equilibration that occurs in one pass through the capillary:(3)

For κ>>1, the venous concentration leaving the tissue (c_v_) is nearly equal to the tissue concentration (c_T_), the clearance (Clr) approaches 1 and the solute is flow limited. The clearance depends on both the intrinsic permeability (PS) and the blood-water partition coefficient. Solutes that have a high intrinsic permeability may be permeability (i.e. diffusion) limited if a large fraction in the blood is solute bound (i.e. large K_bld-wat_).

### Relationship between the anatomic adipose perfusion rate (F) and the "apparent" perfusion rate (F_A_)

The general, diffusion limited, differential equation describing the change in the well mixed tissue concentration (C_T_(t)) produced by a time dependent arterial input (C_A_(t)) is:(4)

where C_V_(t) is the time dependent venous concentration leaving the tissue and c_A _and c_V _are the unbound free blood water concentration. By definition, if the tissue exchange is flow limited, then the venous concentration equilibrates with the tissue concentration (c_v_(t) = c_T_(t)):(5)

Using the definition of clearance (eq. [6]), the general diffusion limited equation (eq. [5]) can be written in the form:(6)

where F_A _is defined as the "apparent" perfusion rate. Equation [6] is identical to the flow limited expression (eq. [5]), the only difference is that F_A _is used in place of the true anatomic perfusion rate (F). Using eq [6], the general differential equation that describes the time dependent adipose tissue concentration (C_T_(t)) as a function of the arterial concentration (C_A_(t)) is then:(7)

The adipose tissue concentration (C_T_) depends on just two parameters - the apparent perfusion rate (F_A_) and the adipose/blood partition coefficient (K_ad-bld_).

### Relationship between K_bld-wat _and octanol/water partition coefficient (K_ow_)

For the solutes used here the experimental value of K_bld-wat _is not available and the following procedure was used to estimate it. It is well recognized that the tissue/blood partition for the highly lipid soluble molecules is roughly equal to the tissue/blood lipid concentration ratio [9-11], indicating that, as a first approximation, solutes in the blood are bound as if they were binding to an equivalent blood lipid (i.e. oil) fraction. Thus, the blood/water concentration can be approximated by:(8)

where K_oil-wat _is the oil (e.g. olive oil)/water partition coefficient and f_L _is the equivalent blood lipid concentration. The last equality in eq. [8] results from the observation that, for non-polar solutes, K_oil-wat _is approximately equal to the octanol/water partition coefficient (K_ow_). A detailed analysis of the dependence of the relationship between K_oil-wat _and K_ow _on chemical structure is described in section 2 of the supplemental file (Additional File 1). Equation [8] is only an approximate estimate of K_bld-wat _since there is clearly some specific binding to the cellular and protein blood components [12,13]. The analysis described below relies on using compounds with a very large range of K_ow _(10^3^) so that small errors in the absolute value of K_bld-wat _are relatively insignificant.

The value of f_L _(= K_bld-wat_/K_oil-wat _= K_bld-oil _≈ 1/K_ad-bld_) can be estimated from the equilibrium partition between blood and adipose tissue (i.e.,oil). In humans, this ratio is about 0.005 for a large series of polychlorinated biphenyls [14]. In addition, in a recent large compilation of K_tis-bld _by deBruyn and Gobas [15] the value of 1/K_ad-bld _is in the range of 0.005 for most of the very highly fat soluble solutes. This value of 0.005 is close to the directly measured values of blood lipid fraction in humans [9] and rats [10] and a value of f_L _= 0.005 will be assumed here. With the exception of PCB 2,2',5,5' (Table 1), all the Oberg et. al. [16] solutes had values of f_L _(= 1/K_ad-bld_) of about 0.005.

**Table 1:** Summary of the experimental data and the model analysis.

Reference	Solute	log K_ow _(ref)	F_A _(kg/min/kg)	K_ad-bld_	Comments
Oberg [16]	PCB 2,4,4'	5.67 [25]	0.15	314	Simultaneous chemical measurements
		
	PCB 2,2',5,5'	5.84 [25]	0.05	89	
		
	PCB 3,3',4,4'	6.36 [25]	0.08	136	
		
	PCB 2,2',3,4,5'	6.29 [25]	0.08	136	
		
	PCB 2,2',4,5,5'	6.38 [25]	0.035	132	
		
	PCB 2,3,3',4,4'	6.65 [25]	0.04	263	
		
	PCB 2,3',4,4',5	6.74 [25]	0.03	226	
		
	PCB 2,2',3,4,4',5	6.83 [25]	0.012	226	
		
	PCB 2,2',4,4',5'	6.92 [25]	0.013	226	
		
	PCB 2,3,3',4,4',5	7.18 [25]	0.015	292	
		
	PCB 2,3,3',4,4',5'	7.18 [25]	0.012	263	
		
	PCB 2,2',3,3,4,4',5	7.27 [25]	0.007	263	
		
	PCB 2,2',3,4,4',5,5'	7.36 [25]	0.005	263	

Muhlebach [23]	PCB 2,2',4,4',5,5'	6.92 [25]	0.02	319	^14^C, Chemical, not metabolized

Ebling [36]	Thiopental	2.85 [37]	0.18	6.5	Chemical

Dallas [38]	Perchloroethylene	3.13 [39]	0.25	124	Chemical

Parham [40]	Dichlorodiphenylsulfone	3.9 (est)	0.12	89	^14^C, low metabolite

Yamaguchi [41]	Hexachlorobenzene	5.46 [42]	0.12	80	Chemical measurements
		
	Hexabromobenzene	6.07 [43]	0.03	27	

Wang [5]	TCDD 2,3,7,8	5.95 [44]	0.02	80	^3^H, low metabolite

Kedderis [35]	TBDD 2,3,7,8	6.5 [44]	0.0024	89	^3^H, low metabolite

Komsta [45]	PCDE 2,2',4,4',5	6.38 [46]	0.015	40	Chemical

For each solute that was modeled, the value of the experimental octanol/water partition coefficient (log K_ow_) and the model values of the "apparent" rat adipose perfusion rate (F_A_) and adipose/blood partition coefficient (K_ad-bld_) are listed. The last column describes the experimental details of the measurements. For "chemical" measurements, the parent solute was directly measured. Tracer (^14^C, ^3^H) measurements of the total (parent and metabolite) concentration were used only if there was independent evidence that the metabolite concentration was negligible.

### Experimental measurement of "apparent" perfusion rate (F_A_)

F_A _was determined from experimental measurements in the rat of the blood and adipose tissue concentration as a function of time. Given the time dependence of the blood concentration entering the tissue and assuming that the tissue is "apparently" flow limited, the adipose tissue concentration depends on only two parameters: F_A _and K_ad-bld _(eq. [7]). A continuous smooth plasma concentration curve was generated from the experimental plasma data points (see additional file 1: section I for details) and used as the arterial input (C_A_(t)). Using this time dependent arterial input, the standard flow limited organ equations (eq. [7]) were solved for the adipose concentration as a function of time. The two adipose parameters F_A _and K_ad-bld _were then adjusted to give the optimal fit to the experimental adipose concentration measurements. In most cases the experiments were carried out for long enough times (up to 132 days) that the adipose tissue had come close to equilibrating with the plasma and K_ad-bld _could be determined directly from this equilibrium value.

The numerical calculations were obtained using PKQuest_Java [17], a freely distributed PBPK software routine that can be downloaded from http://www.pkquest.com. In addition, the free file "Persistent organics" contains the complete experimental data sets for most of the solutes discussed here and a detailed tutorial for reproducing these calculations.

### Value of the anatomic adipose perfusion rate (F)

The determination of PS from eq. [9] requires an estimate of the anatomic adipose perfusion rate (F). The reported values for F in rat adipose tissue vary over a large range depending on methodologies, age, strain and condition (i.e. conscious or anesthetized). In addition, there are large differences in blood flow at different anatomic locations, varying from about 0.15 for epididymal to 0.55 kg/min/kg for mesenteric fat [18,19]. Most recent studies using labeled microspheres in conscious rats report values in the range of 0.18 to 0.25 kg/min/kg [20-22]. A value for F of 0.2 kg/min/kg was assumed in the following calculations.

### Experimental data

The experimental data was obtained from the literature. The central results are those of Oberg et. al. [16] who simultaneously measured the rat plasma and adipose tissue concentration following an oral dose of a mixture of 13 different polychlorinated biphenyls, varying from 3 to 7 substituted chlorines with log K_ow _varying from 5.67 to 7.36 (table 1). These simultaneous chemical measurements represent the ideal data set for this analysis and this is the only measurement of this type that I am aware of.

In addition to the Oberg et. al. data set, the literature was screened and a number of other solutes were also modeled. One important experimental limitation for many solutes is that only the total C^14 ^or H^3 ^labeled equivalent was measured and the parent and metabolite compounds were not distinguished. Some of these tracer measurements were used in this analysis (see table 1) if there was supporting information that the labeled metabolite concentration in the plasma and tissue is relatively low. All of the experimental data that was used, along with comments about the experimental limitations, are summarized in table 1.

For published data that was available only in graphical form, the values were read off the graph using the program UN-SCAN-IT (version 6.0, Silk Scientific Corporation).

### Summary of procedure used to estimate the adipose-blood diffusion limitation (Clr) and the intrinsic adipose capillary permeability-surface area product (PS)

Experimental measurements of the rat arterial blood (C_A_(t)) and adipose tissue (C_T_(t)) concentrations as a function of time were obtained from the literature. From a numerical solution of the differential eq. [7], the values of the two parameters F_A _and K_ad-bld _that gave the best agreement between the predicted and experimental adipose tissue concentration is then obtained. From the ratio of F_A_/F, the value of the adipose clearance (Clr) and the parameter κ for a given solute can be obtained from eqs. [3] and [6]. Using the relationship between K_bld-wat _and K_ow _(eq. [8], κ can be expressed in terms of K_ow_:(9)

As a first approximation, the value of the intrinsic PS (and β) for a given organ and species can be regarded as a constant, independent of the solute. This is because PS is proportional to the aqueous diffusion coefficient which is roughly proportional to 1/radius (Stokes-Einstein relation). For all the solutes considered here the molecular radius varies be less than a factor of 2, a variation that is negligible compared to the variations in K_ow _(≈ 10^3^). Thus, assuming that β is constant, measurements of κ for a series of solutes with a wide range of values of K_ow _can be used to estimate the value of PS using the known values of f_L _(= 0.005) and F (0.2 kg/min/kg).

## Results

**Figure 2:**
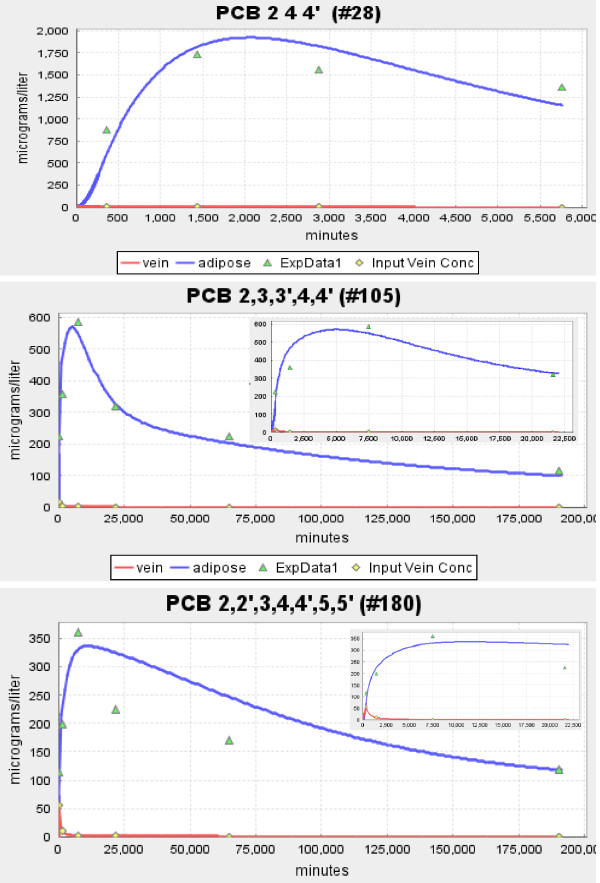
**Absolute plot of the time dependence of the experimental adipose (green triangle) and blood (yellow diamond) concentration and the model predictions for adipose (blue line) and blood (red line)**. The three panels show the results of Oberg et. al. [16] for a PCB with low (log K_ow _= 5.67), moderate (log K_ow _= 6.65) and high (log K_ow _= 7.36) octanol/water partition coefficient. The insets show the results at short times.

**Figure 3:**
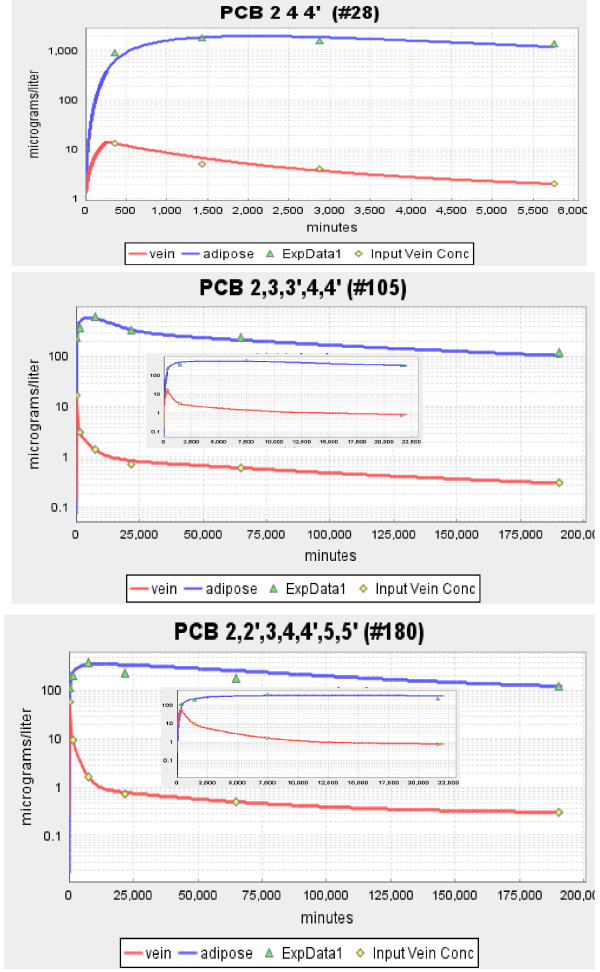
**Semi-log plot of the time dependence of the experimental adipose (green triangle) and blood (yellow diamond) concentration and the model predictions for adipose (blue line) and blood (red line)**. The three panels show the results of Oberg et. al. [16] for a PCB with low (log K_ow _= 5.67), moderate (log K_ow _= 6.65) and high (log K_ow _= 7.36) octanol/water partition coefficient. The insets show the results at short times.

The three panels in Figures 2 and 3 show the model fits to the experimental data of Oberg et. al. [16] for the solutes with the lowest (2,4,4' PCB, log K_ow _= 5.67), intermediate (2,3,3',4,4' PCB, log K_ow _= 6.65) and highest (2,2',3,4,4',5,5', log K_ow _= 7.36) value of K_ow_. Figure 2 shows the absolute plot and the insets shows the early time data. Figure 3 shows the semi-log plot of the same data. The red line is the smoothed fit to the plasma data which is used as the arterial input concentration to the organ. For all the solutes investigated, this smoothed curve provided a nearly perfect fit to the experimental blood concentration data points.

**Figure 4:**
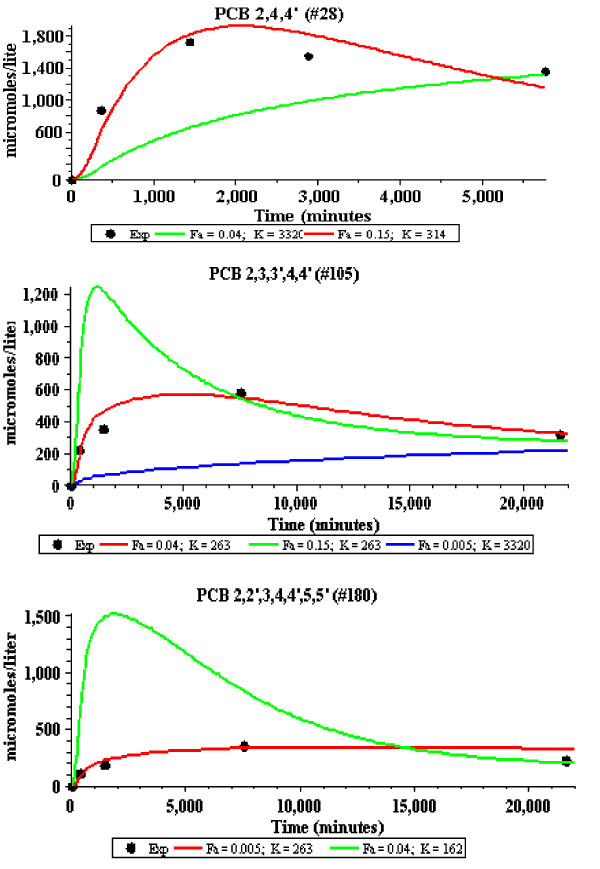
**Comparison of the model fits to the adipose concentrations as a function of the "apparent" perfusion rate (F_A_) for the three solutes described in Figures 1 and 2**. For each solute, the optimal value of F_A _for that solute (red line) is compared with the optimal fit that can be obtained using the value of F_A _for the other solutes (green and blue line). For the green and blue fits, the value of the adipose/blood partition (K) was adjusted to approximately fit the last time point. The value of F_A _and K for each line is listed at the bottom of each panel.

The model fit to the adipose concentration data points is a function of two parameters: F_A_, and K_ad-bld_. These two parameters were adjusted to give the optimal fit to the adipose data, and these are the results that are shown in Figures 2 and 3 and are listed in Table 1. The adipose tissue fits are clearly not perfect. However, because of the large qualitative difference in the kinetics for the 3 solutes shown in Figure 2, there is no question that there are significant differences in the corresponding values of F_A_. This is dramatically illustrated in Figure 4 where the plots of the optimal values of F_A _are compared with the best fits that can be obtained using the value of F_A _for the neighboring solute in Figure 2. For example, in the middle panel of Figure 4, the red line shows the optimal fit (F_A _= 0.04) to the experimental adipose concentration data points for PCB 2,3,3',4,4' (solid circles). The green line is the predicted fit if the F_A _were equal to that of PCB 2,4,4' (F_A _= 0.15) and the blue line is the optimal fit if the F_A _was that of PCB 2,2',3,4,4',5,5' (F_A _= 0.005). The values of K_ad-bld _for the green and blue lines have been adjusted so that the curves fit the long time (i.e. equilibrium) data point.

**Figure 5:**
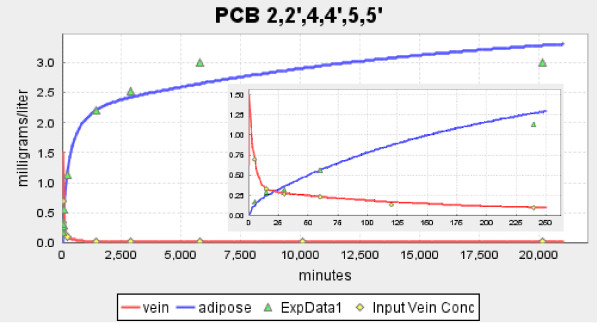
**Absolute plot of the time dependence of the experimental adipose (green triangle) and blood (yellow diamond) concentration and the model predictions for adipose (blue line) and blood (red line) for the PCB 2,2',4,4',5,5' data of Muhlebach et. al**. [23]. The insets show the results at short times.

**Figure 6:**
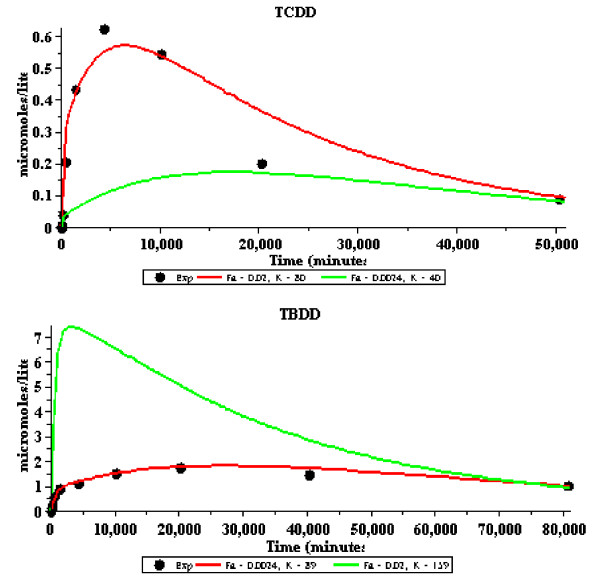
**Perfusion rate dependence (F_A_) of the model fits to the experimental adipose concentration measurements (solid circles) for tetrachlorodibenzo-p-dioxin (TCDD, Wang et. al**. [5]**) and tetrabromodibenzo-p-dioxin (TBDD, Kedderis et. al**. [35]**)**. The red line is the predicted fit using the optimal value of F_A _for that solute and the green line is the fit using the F_A _of the other solute. For the green lines, the value of the adipose/blood partition (K) was adjusted to approximately fit the last time point.

Table 1 lists the model values for F_A _and K_ad-bld _for the 13 PCBs studied by Oberg et. al. [16] along with a number of other solutes. One limitation of the Oberg et. al. data is that the first data point is at 6 hours and the early time adipose kinetics are missed. To address this, results are shown in Figure 5 for the very slowly metabolized PCB 2,2',4,4',5,5' which has been carefully investigated by Muhlebach et. al.[23] and has adipose data points at early times (5, 15, 30, 60 and 240 minutes). This data (including the early times) is well described by an F_A _of 0.02, which is in good agreement with the Oberg. et. al. data for PCBs with a similar K_ow_. Three of the solutes in Table 1 (thiopental, perchloroethylene and dichlorodiphenylsulfone) have low values of K_ow _(and K_bld-wat_) and would be expected to be flow limited. Also listed in Table 1 are two pairs of solutes that are in the same chemical class and used similar experimental measurements. The first pair isTCDD and TBDD, for which both the pharmacokinetic and K_ow _measurements were made by the same laboratory. The second is the hexachlorobenzene and hexabromobenzene pair whose kinetics were described in the same publication. For both of these pairs, there is a significant decrease in the values for F_A _with an increase in K_ow_. Figure 6 compares the model F_A _dependence for TCDD and TBDD, similar to the plots in Figure 4.

**Figure 7:**
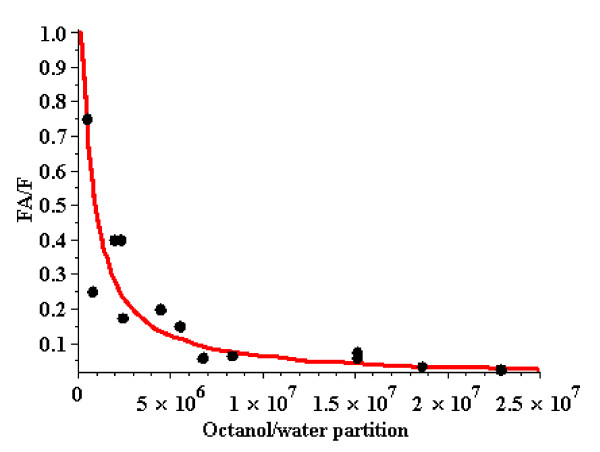
**Plot of the degree of diffusion limitation (F_A_/F) as a function of the octanol/water partition coefficient for the 13 PCBs studied by Oberg et. al**. [16] (see Table 1 for numerical values). The red line is a plot of eq. [9] with a β (eq. [9]) of 0.75 × 10^6^, adjusted to provide the best fit to the data.

The crucial test of this model of diffusion limitation is to determine if the fractional clearance (= Clr = F_A_/F) is described by eq. [9] Figure 7 shows the plot of the Oberg et. al. [16] values of F_A_/F (solid circles) as a function of K_ow_. The red line is the plot of 1-exp(-β/K_ow_), with β (eq. [9]) adjusted to provide the best fit to the Oberg et. al. data. For the value of β (= 0.75 × 10^6^) that provides the best fit to the data in Figure 6, PS = βf_L_F = 750 min^-1 ^(assuming f_L _= 0.005 and F = 0.2).

## Discussion

As described by eq. [3], the blood-water partition coefficient (K_bld-wat_) has a critical role in determining whether tissue-solute exchange is diffusion limited. The capillary permeability limitation produced by this blood lipid solute binding is directly analogous to the carbon monoxide (CO) pulmonary diffusion limitation that results from the high affinity of hemoglobin for CO [24]. This same effect was also invoked to quantitate the capillary diffusion limitation of albumin bound β-lactam antibiotics [3]. As seen in eq. [3], given a finite intrinsic permeability (PS), the solute must become diffusion limited if K_bld-wat _is large enough. The only question is at what value of K_bld-wat _does the solute become diffusion limited?

To answer this question, the degree of diffusion limitation was determined for a series of solutes with varying values of octanol/water partition coefficient (K_ow_). The "diffusion limitation" was quantitated in rat adipose tissue from experimental measurements of the ratio of the "apparent" perfusion rate (F_A_) relative to the anatomic perfusion rate (F). Assuming that K_bld-wat _is roughly proportional to K_ow _(eq. [8]), one should observe an increase in diffusion limitation as K_ow _increases. One would expect that this relationship would only be roughly satisfied because the exact relationship between K_ow _and K_bld-wat _should depend on the specific blood protein binding and the detailed chemical structure [12,13]. This is the reason that it is especially important to have the Oberg et. al. [16] experimental data set for a large series of solutes with similar chemical structures and with K_ow _varying over a 50 fold range. Another factor that introduces variations in the relation between diffusion limitation and K_ow _is that there are large variations in the experimental measurements of K_ow_, depending on the experimental technique that is used. An advantage of the Oberg et. al. data set is that the values of K_ow _for all the solutes was determined using the same approach [25]. As shown in Table 1, for the Oberg et. al. data there is a qualitative increase in the degree of diffusion limitation (i.e. reduction in "apparent" perfusion rate F_A_) as K_ow _increases. Quantitatively, this diffusion limitation is quite will described by eq. [9] (Figure 7).

Also listed in Table 1 are the values of F_A _for some other solutes. Three of these solutes (thiopental, perchloroethylene, and dichlorodiphenylsulfone) have very small values of K_ow _and one would expect them to be flow limited. Consistent with this prediction, their values of F_A _are close to the assumed anatomic value (F) of 0.2 kg/min/kg.

Results are also shown in table 1 for hexachlorobenzene, hexabromobenzene, TCDD, TBDD and PCDE. The hexachlorobenzene and hexabromobenzene pair are directly comparable since their kinetics were described in the same publication using identical procedures. Similarly, the TCDD and TBDD pair results were carried out by the same lab using similar procedures. For both of these solute pairs with very similar structures, there is a significant increase in diffusion limitation (i.e. decrease in F_A_) with an increase in K_ow _(see also Figure 6 for a sensitivity analysis of the TCDD and TBDD results). These five solutes have values of K_ow _in the same range as the flow limited solutes studied by Oberg et. al. [16] and, qualitatively, have a similar increase in diffusion limitation (decrease in F_A_) with increasing K_ow_. Quantitatively, these solutes do not fall on exactly the same F_A_/F versus K_ow _curve (Figure 7) that was obtained for the Oberg solutes. As discussed above, this is not surprising because one would predict that the proportionality between K_ow _and K_bld-wat _should have some solute dependence and should also depend on the specific details of, for example, the K_ow _measurements.

From the plot of F_A_/F versus K_ow _for the Oberg et. al. data (Figure 7), one can estimate that the corresponding value of the "intrinsic" PS for rat adipose tissue is about 750/min (see Results). To put this very large value in perspective, the highest capillary permeability that has been directly measured is a PS of about 1/min for Na^+ ^in heart capillaries [26]. Solutes with a PS of 750/min only become diffusion limited because they have a very large value of K_bld-wat _(≈ 5000).

One can use this value of PS to estimate the "equivalent" thickness of the rat blood-adipose diffusion limiting barrier. The permeability is equal to:(10)

where D_W _is the aqueous diffusion coefficient and W is the thickness. Thus, relating W to PS:(11)

where is S is the rat adipose capillary surface area. Using values of 5 × 10^-6 ^cm^2^/sec [27] for PCB D_W _and 35 cm^2^/cm^3 ^[28] for rat S, the value of W is about 1.4 × 10^-5 ^cm for a PS of 750/min. This value for W (0.14 μm) is approximately equal to the thickness of the rat adipose capillary endothelium (0.25 μm) [29] and seems a reasonable estimate of the aqueous diffusive barrier between the blood (where the solutes are well-stirred and bound to albumin and the lipoproteins) and the adipose lipid.

An inherent assumption in this analysis is that the rate limiting step is the aqueous diffusion across the capillary wall and that the diffusion in the capillary blood and adipose tissue is not limiting. Diffusion in the blood should not be rate limiting because it is well stirred and the solute is bound at a high concentration relative to the water. The relative rates of diffusion in the aqueous and fat tissue are described by:(12)

and the ratio of lipid to aqueous diffusion is:(13)

Since the lipid (i.e. olive oil) viscosity is about 85 times greater than water, the Stokes-Einstein relation predicts that the lipid diffusion coefficient is about 85 times smaller than in water. However, this relation is valid only if the solute is much larger than the solvent, which is not true for diffusion in olive oil. For small solutes, such as O_2 _or N_2_, the diffusion coefficient in olive oil is only about 3 times smaller than in water [30]. Rogacheva et. al. [31] measured diffusion coefficients of 2-nonanone and benzaldehyde in oil that were about 10 times less than the water value. As a rough estimate, it is assumed that the diffusion coefficient in olive oil for the solutes studied here is about 20 times less than in water (D_L_/D_W _= 0.05). Since K_oil-wat _for the solutes considered here is 10^5 ^or greater, aqueous diffusion (eq. 13) is clearly the rate limiting step.

A novel feature of this analysis is the approach that was used to determine the "apparent" adipose perfusion. In the previous publications from which this experimental data was obtained, the blood and adipose concentrations were simultaneously modeled using the complete PBPK multi-tissue model. In many cases the model fit to the blood concentration is only poorly approximated by this PBPK model, presumably because of errors in the model assumptions (e.g. non-linearity). In contrast, in this analysis the exact experimental blood concentration is fit by a smooth curve so that the model input to the adipose tissue is identical to the experimental arterial concentration. The adipose perfusion rate is then adjusted to give the best fit to the experimental adipose concentration. This provides a significantly more accurate estimate of adipose perfusion then the total PBPK model fit with the incorrect blood concentration.

It is of interest to try to extrapolate these rat results to humans. The degree of diffusion limitation for a given K_ow _is determined by the parameter β = PS/(f_L_F) (eq. [9]). P corresponds to the diffusion limiting aqueous layer and should be similar for rat and human. Since rats [32] and humans [33] have similar adipose capillary densities, they should have similar capillary surface areas (S). Also, the blood lipid fraction (f_L_) is similar for rats and humans. One factor that does differ between rats and humans is the adipose blood flow (F), with the average human value of about 0.05 kg/kg/min [34] about 1/4 the rat value (0.2 kg/kg/min). Thus, the human value for β is about 4 times larger than the rat value and there should be corresponding less diffusion limitation for the same value of K_ow_.

## Conclusions

Although it has been previously recognized that some highly fat soluble persistent organic chemicals are diffusion limited [5-7], this analysis provides the first physical explanation of this diffusion limitation along with its quantitative dependence on K_ow_. This diffusion limitation follows directly from the basic physiology of the blood tissue exchange. There must be some finite aqueous unstirred layer between the blood and the adipose lipid and this layer will become rate limiting if K_bld-wat _is large enough. The results described here show that this diffusion limitation reduces the apparent rat adipose perfusion rate from the anatomic value of about 0.2 kg/min/kg for the flow limited solutes (log K_ow _< 5) to a value of about 0.005 kg/min/kg for a PCB with a log K_ow _of 7.36. The thickness of the limiting layer estimated from this analysis is about 0.14 μm, approximately equal to the thickness of the adipose capillary epithelial cell.

## List of abbreviations

PBPK: Physiologically based pharmacokinetics; F: anatomic organ perfusion rate (kg/min/kg); F_A_: "apparent" perfusion rate assuming the organ kinetics are flow limited; a: capillary radius (cm); L: capillary length (cm); N: capillary density (#/cm^3^); S: 2πaNL: capillary surface area surface area per cm^3 ^tissue (1/cm); P: intrinsic permeability (cm/min); PS: intrinsic organ permeability-surface area product (min^-1^); Clr: fraction of solute that equilibrates across capillary in one pass; C_i_: total concentration (mole/kg) in organ i. i: B(blood), A(artery),V(vein), T(adipose); c_i_: free concentration in water (moles/cm^3^); f_L_: "equivalent" lipid fraction of blood; K_bld-wat_: blood/water partition coefficient; K_ad-wat_: adipose/water partition coefficient; K_ad-bld_: adipose/blood partition coefficient; K_oil-wat_: oil/water partition coefficient; K_ow_: octanol/water partition coefficient; D_w _and D_L_: Diffusion coefficient in water and lipid (cm^2^/sec); W: thickness (cm) of equivalent aqueous layer corresponding to intrinsic permeability; PCB: polychlorinated biphenyl; PCDE: pentachlorodiphenyl ether; TCDD: tetrachlorodibenzo-p-dioxin; TBDD: tetrabromodibenzo-p-dioxin.

## Competing interests

The author declares that they have no competing interests.

## Authors' contributions

DGL is entirely responsible for the contents of this paper and has read and approved the final manuscript.

## Pre-publication history

The pre-publication history for this paper can be accessed here:

http://www.biomedcentral.com/1472-6904/10/1/prepub

## Supplementary Material

Additional file 1 I. Mathematical representation of the time dependent plasma concentration.
II. Prediction of oil/water partition coefficient using octanol/water coefficient. Click here for file
