# Supplementary material for: Quantitative relationship between the octanol/water partition coefficient and the diffusion limitation of the exchange between adipose and blood
Source: BMC Clin Pharmacol. 2010 Jan 7;10:1. doi: 10.1186/1472-6904-10-1 (PMC2845558; doi:10.1186/1472-6904-10-1)
Supplement: Additional file 1 — I. Mathematical representation of the time dependent plasma concentration. II. Prediction of oil/water partition coefficient using octanol/water coefficient. [file 1472-6904-10-1-S1.DOC]

**Additional File 1**

**I. Mathematical representation of the time dependent plasma concentration.**

The procedure used to determine the “apparent” organ perfusion rate was to fit the experimental plasma concentration data points with a smooth function and then determine the perfusion rate that gave the best fit to the experimental adipose tissue concentration for this arterial input. The smooth plasma function was determined as follows. It was assumed that the plasma concentration (C(t)) resulted from a constant input of duration T into a 1, 2 or 3 compartment system:

$$C(t) = \int_{0}^{t} A*[1-H(\tau-T)] rfunc(\tau) d\tau$$

Where A is the constant infusion rate, H is the Heaviside unit step function, and rfunc is the response function for a delta input to a 1, 2 or 3 compartment system. For example, for 2 compartments, the response function is.

$$rfunc(t) = a_{1}\exp(-t/a_{2}) + a_{3}\exp(-t/a_{4})$$

In addition, a constant term (a_0_) was added to the C(t). For example, for 2 compartments, the final expression for C(t) has 5 adjustable parameters (a_0_ .. a_4_):

C(t) = a_0_ – A[-a_1_a_2_+a_1_a_2_exp(-t/a_2_)+a_1_a_2_H(t-T)-a_1_a_2_H(t-T)exp((-t+T)/a_2_
 -a_3_a_4_+a_3_a_4_exp(-t/a_4_)+a_3_a_4_H(t-T)exp((-t+T) /a_4_)]

Non-linear Powell minimization was used to find the parameter set that gave the best fit to the experimental plasma concentration. The procedure that was used to find the parameters was to fit the plasma concentration sequentially to 1, then 2, then 3 compartments, using the parameters from the previous lower order fit as starting values for the higher compartment fit.

Some of the experiments used intravenous input for which the value of T = 0.5 minutes was assumed, while others used an oral input for which T = 260 minutes was assumed.

**II. Prediction of oil/water partition coefficient using octanol/water coefficient.**

For most of the solutes discussed in the main text, there are no available experimental values of the oil/water (e.g. olive oil/water) partition coefficient. However, it will be shown here that for solutes with these relatively simple structures one can predict (within a factor of about 2) the oil/water partition coefficient from the experimental values of the octanol/water partition coefficient. Figure 1 (below) shows a plot of (log octanol/water - log oil/water partition) versus the log octanol/water partition for a series of non-polar solutes. Figure 2 (below) shows a similar plot for solutes with 1 or 2 aliphatic or aromatic hydroxyl groups and no other polar groups. The oil/water data in these figures is based on values from the literature [1-12]. The octanol/water data are the recommended values from the web site of James Sangster (<http://logkow.cisti.nrc.ca/logkow/intro.html>).

It can be seen in fig. 1 that for the non-polar solutes, the oil-water and octanol-water partition are nearly identical (octanol-water partition about 25% less then oil-water) over a very wide range of structures and octanol/water partition coefficient. As shown in fig. 2, the addition of 1 aliphatic hydroxyl makes the oil-water partition about 1.03 log units (factor of 10.5) smaller than the octanol-water partition and one aromatic hydroxyl makes the oil-water about 0.82 log units (factor 6.6) smaller. The larger partition of the hydroxyl compounds in octanol versus olive oil results from the hydrogen bond forming potential of octanol. The data in fig. 2 also show that the effect of adding additional hydroxyls is roughly additive.

**References**

1. Chiou CT: **Partition coefficients of organic compounds in lipid-water systems and correlations with fish bioconcentration factors.** *Environ Sci Technol* 1985, **19:**57-62.

2. Jabusch TW, Swackhamer DL: **Partitioning of polychlorinated biphenyls in octanol/water, triolein/water, and membrane/water systems.** *Chemosphere* 2005, **60:**1270-1278.

3. Gargas ML, Burgess RJ, Voisard DE, Cason GH, Andersen ME: **Partition coefficients of low-molecular-weight volatile chemicals in various liquids and tissues.** *Toxicol Appl Pharmacol* 1989, **98:**87-99.

4. Meulenberg CJ, Wijnker AG, Vijverberg HP: **Relationship between olive oil:air, saline:air, and rat brain:air partition coefficients of organic solvents in vitro.** *J Toxicol Environ Health A* 2003, **66:**1985-1998.

5. Kaneko T, Wang PY, Sato A: **Partition coefficients of some acetate esters and alcohols in water, blood, olive oil, and rat tissues.** *Occup Environ Med* 1994, **51:**68-72.

6. Oldendorf WH: **Lipid solubility and drug penetration of the blood brain barrier.** *Proc Soc Exp Biol Med* 1974, **147:**813-815.

7. Fiserova-Bergerova V, Tichy M, Di Carlo FJ: **Effects of biosolubility on pulmonary uptake and disposition of gases and vapors of lipophilic chemicals.** *Drug Metab Rev* 1984, **15:**1033-1070.

8. Sato A, Nakajima T: **Partition coefficients of some aromatic hydrocarbons and ketones in water, blood and oil.** *Br J Ind Med* 1979, **36:**231-234.

9. Sato A, Nakajima T: **A structure-activity relationship of some chlorinated hydrocarbons.** *Arch Environ Health* 1979, **34:**69-75.

10. Eger EI, 2nd, Ionescu P, Laster MJ, Gong D, Hudlicky T, Kendig JJ, Harris RA, Trudell JR, Pohorille A: **Minimum alveolar anesthetic concentration of fluorinated alkanols in rats: relevance to theories of narcosis.** *Anesth Analg* 1999, **88:**867-876.

11. Zhang Y, Trudell JR, Mascia MP, Laster MJ, Gong DH, Harris RA, Eger EI, 2nd: **The anesthetic potencies of alkanethiols for rats: relevance to theories of narcosis.** *Anesth Analg* 2000, **91:**1294-1299.

12. Leo A, Hansch C, Elkins D: **Partition coefficients and their uses.** *Chemical Reviews* 1971, **71:**525-616.


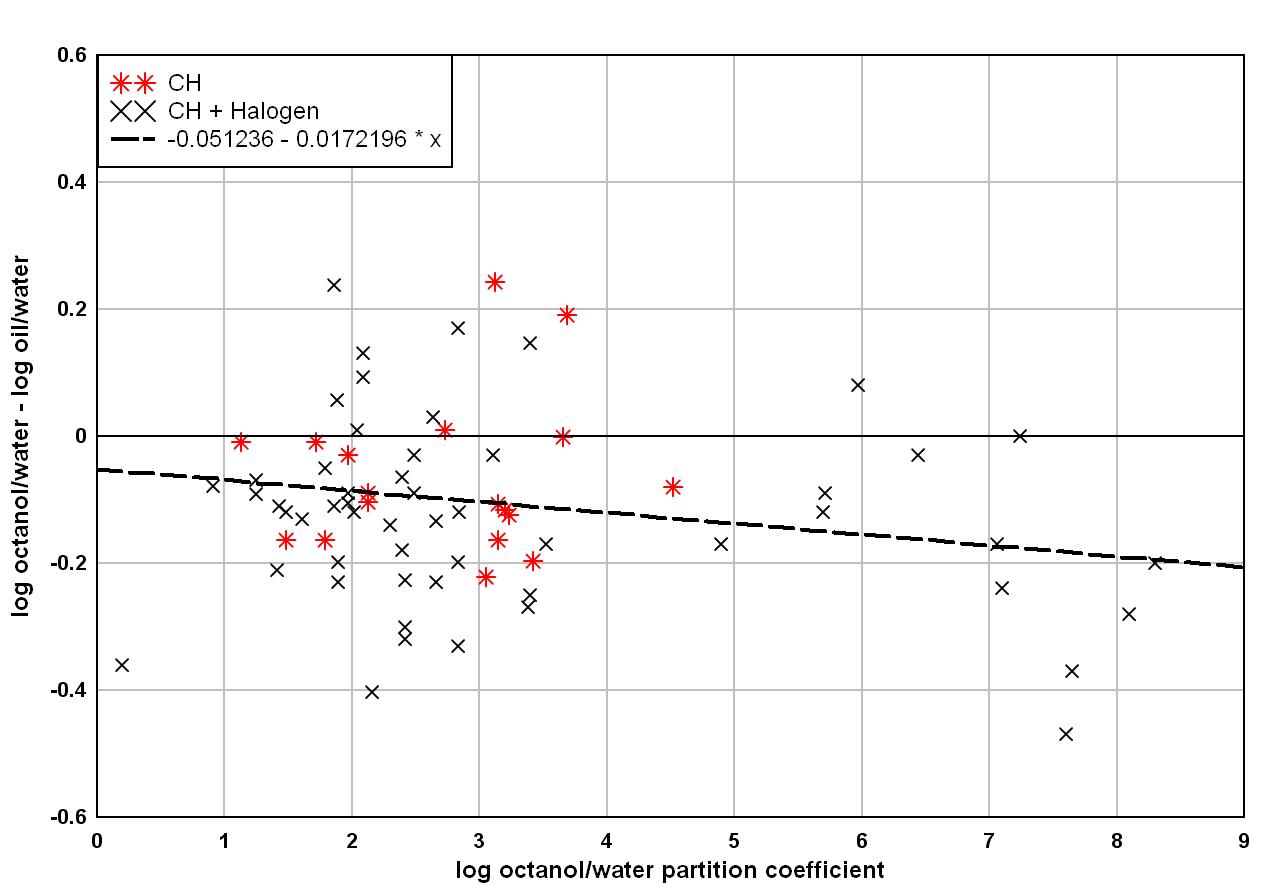


Figure 1. Plot of (log octanol/water – log oil/water) versus log octanol/water for non-polar solutes. Pure hydrocarbon molecules (*) and halogenated hydrocarbons (x) are indicated. The dashed line is the least square fit to all the data.


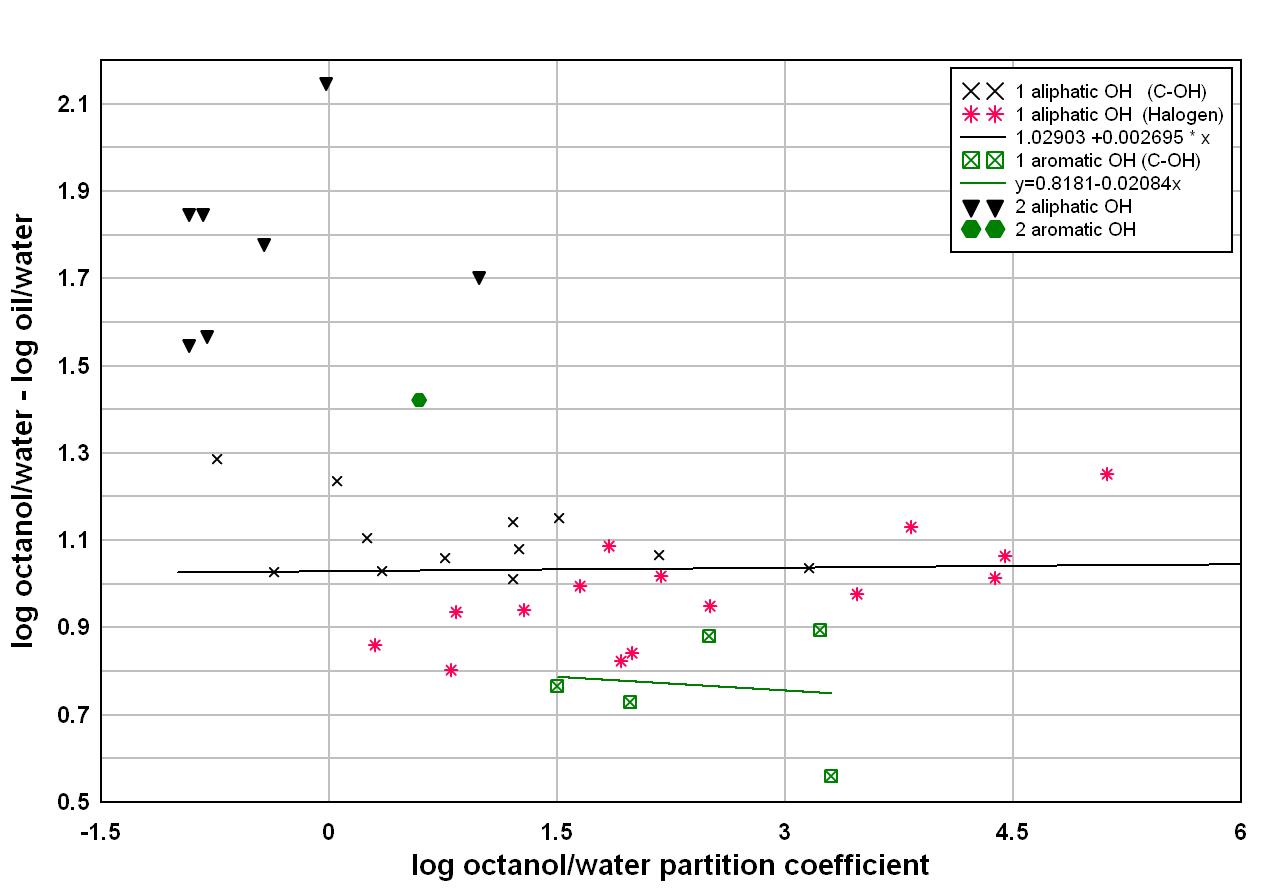


Figure 2. Plot of (log octanol/water – log oil/water) versus log octanol/water for solutes with 1 or 2 hydroxyl groups.
